# Guillain-Barre syndrome presenting with sensory disturbance following a herpes virus infection: a case report

**DOI:** 10.1186/1752-1947-5-563

**Published:** 2011-12-04

**Authors:** Fotinie Ntziora, Athina Euthimiou, Maria Tektonidou, Anastasios Andreopoulos, Kostas Konstantopoulos

**Affiliations:** 1 University of Athens School of Medicine, First Department of Medicine, Laiko General Hospital, Athens, Greece; 2 Department of Neurology, Laiko General Hospital, Athens, Greece

## Abstract

**Introduction:**

We present a case of an unusual clinical manifestation of Guillain-Barre syndrome following a pre-existing herpes virus infection. Although there have been several reports describing the co-existence of herpes virus infection and Guillain-Barre syndrome, we undertook a more in-depth study of the cross-reactivity between herpes viruses and recommend a follow-up study based on serology tests.

**Case presentation:**

A 39-year-old healthy Caucasian man with Guillain-Barre syndrome presented to our facility initially with sensory disturbance, followed by an atypical descending pattern of clinical progression. On physical examination, our patient showed hot and cold temperature sensory disturbance under the T4 vertebrae level, symmetrically diminished muscle power mainly to his lower limbs, blurred vision, a loss of taste and paresis and diminished reflexes of his lower limbs. Serology test results for common viruses on hospital admission were positive for cytomegalovirus immunoglobulin M, cytomegalovirus immunoglobulin G, herpes simplex virus immunoglobulin M, herpes simplex virus immunoglobulin G, Epstein-Barr virus immunoglobulin M, and varicella zoster virus immunoglobulin G, borderline for Epstein-Barr virus immunoglobulin G and negative for varicella zoster virus immunoglobulin M. At one month after hospital admission his test results were positive for cytomegalovirus immunoglobulin M, cytomegalovirus immunoglobulin G, herpes simplex virus immunoglobulin G, Epstein-Barr virus immunoglobulin G, varicella zoster virus immunoglobulin G, borderline for herpes simplex virus immunoglobulin M and negative for Epstein-Barr virus immunoglobulin M and varicella zoster virus immunoglobulin M. At his six month follow-up, tests were positive for cytomegalovirus immunoglobulin G, herpes simplex virus immunoglobulin M, herpes simplex virus immunoglobulin G, Epstein-Barr virus immunoglobulin G and varicella zoster virus immunoglobulin G and negative for cytomegalovirus immunoglobulin M, Epstein-Barr virus immunoglobulin M and varicella zoster virus immunoglobulin M.

**Conclusions:**

The clinical manifestation of Guillain-Barre syndrome in our patient followed a combined herpes virus infection. The cross-reactivity between these human herpes viruses may have a pathogenic as well as evolutionary significance. Our patient showed seroconversion at an early stage of Epstein-Barr virus immunoglobulin M to immunoglobulin G antibodies, suggesting that Epstein-Barr virus might have been the cause of this syndrome. Even if this case is not the first of its kind to be reported, it may contribute to a better understanding of the disease and the cross-reaction mechanisms of herpes virus infections. This case report may have a broader clinical impact across more than one area of medicine, suggesting that cooperation between different specialties is always in the patient's best interest.

## Introduction

Guillain-Barre syndrome (GBS) is an acute polyradiculoneuropathy marked by flaccid areflexic paralysis. Although GBS has been viewed as a unitary disorder, it is widely accepted that it includes distinctive subtypes, such as acute inflammatory demyelinating polyradiculoneuropathy, acute motor axonal neuropathy, acute motor-sensory axonal neuropathy and Miller-Fisher syndrome, amongst others [1,2]. Both sexes are equally affected; healthy adults are more commonly affected than children. The onset of the symptoms of sudden and severe paralysis may occur, but the overall prognosis is good, with approximately 85% of survivors making a good functional recovery. Early diagnosis and progress in methods of support have decreased mortality rates and improved outcome.

GBS is considered a post-infectious, immune-mediated disease. Symptoms of a preceding upper respiratory tract infection or gastrointestinal infection are often reported prior to the onset of GBS symptomatology. *Campylobacter jejuni*, cytomegalovirus (CMV), Epstein-Barr virus (EBV) and *Mycoplasma pneumoniae *have been identified as the predominant causes associated with GBS. The type of antecedent infection in GBS is related to specific serum anti-ganglioside antibodies and clinical subgroups. Many of the identified infectious agents may induce antibody production against specific gangliosides and glycolipids, such as GM1 and GD1b, distributed throughout the myelin in the peripheral nervous system. Molecular mimicry between infectious agents and gangliosides plays an important role in inducing these antibodies [3,4].

## Case presentation

A 39-year-old Caucasian man was referred to our emergency department due to a sudden loss of hot and cold sensation when taking a shower, followed by a progressive instability and weakness of his lower extremities. Our patient also complained of diplopia, dim vision and circumoral numbness. Our patient reported an upper respiratory tract infection for which he was being treated with an antibiotic (macrolide). Neurologic symptomatology began a few days later. His medical history was unremarkable apart from seasonal asthma. He used to smoke 20 cigarettes per day and drink alcohol only on social occasions. He was the father of two children. No high-risk sexual behavior was reported.

On physical examination, he had hot and cold temperature sensory disturbance under the T4 vertebrae level, symmetrically diminished muscle power mainly to his lower limbs, blurred vision and a loss of taste. Further neurological examination revealed paresis and diminished reflexes of his lower limbs; mainly the jerk ankle and patellar reflex. Cerebral pathology results following an MRI scan were negative. However, our patient reported a loss of balance, a failure to stand or walk and numbness. In summary, our patient showed progressive weakness of his lower limbs due to neuropathy, areflexia, sensory involvement and cerebella ataxia, and with the duration of the disease being less than four weeks, the required and some of the supportive diagnostic criteria for GBS were met. Other physical examinations were normal.

Two lumbar punctures were performed, the first on admittance and the second 14 days following commencement of the symptoms (Table 1). The diagnostic tests are summarized chronologically in Table 2. Serology tests for neurotropic viruses were requested for differential diagnosis purposes. Hepatitis, human immunodeficiency virus (HIV) and varicella zoster virus (VZV) test results were negative, as were results for West Nile virus (WNV) with indirect enzyme-linked immunosorbent assay (ELISA) immunoglobulin G (IgG) and M antibody capture (MAC)-ELISA (IgM). However, CMV, herpes simplex virus (HSV) and EBV IgM antibody test results were positive. Blood CMV antigen and polymerase chain reaction (PCR) tests for CMV were negative, as was the PCR test for CMV from the cerebrospinal fluid (CSF). Blood, urine and CSF cultures were sterile. A tuberculin skin test and CSF culture for tuberculosis (TBC) were negative. Serology tests were repeated one month after hospital admittance and at a six month follow-up appointment. The comparative results are summarized in Table 2.

**Table 1:** Comparison of the two lumbar puncture (LP) results from cerebrospinal fluid (CSF)

Result	First LP	Second LP
Cells per mm^3^	5	20

Glucose (mg/dL)	75	64

Protein (g/dL)	0.30	1

Lactate dehydrogenase (U/L)	51	56

Gram staining	Negative	Negative

Cryptococcus	Negative	Negative

PCR CMV	Negative	-

CMV = cytomegalovirus; PCR = polymerase chain reaction.

**Table 2:** Diagnostic tests and their results in chronological order (blood)

Diagnostic test	First day	First month	Six months
CMV IgM	+	+	-

CMV IgG	+	+	+

HSV IgM	+	Borderline	+

HSV IgG	+	+	+

EBV IgM	+	-	-

EBV IgG	Borderline	+	+

VZV IgM	-	-	-

VZV IgG	+	+	+

WNV IgM	-	NA	NA

WNV IgG	-	NA	NA

VDRL	-	NA	NA

HIV	-	NA	NA

HBV	-	NA	NA

HCV	-	NA	NA

CMV Ag	-	NA	NA

PCR CMV	-	NA	NA

CMV Ag = cytomegalovirus antigenemia; HBV = hepatitis B virus; HCV = hepatitis C virus; HSV = herpes simplex virus; NA = not applicable; PCR = polymerase chain reaction; VDRL = Venereal Disease Research Laboratory; VZV = Varicella zoster virus; WNV = West Nile virus.

Results of a chest X-ray were negative. A brain MRI scan showed no abnormalities of the cerebral parenchyma, but a slight inflammation of the ethmoid and right mastoid sinus was noted. In view of the neurological findings, his jugular and lumbar spinal column were scanned. No abnormalities except degenerative lesions and slightly slipped disks were noted, but these findings were irrelevant to the clinical symptoms. Brain, lung and abdominal computed tomography showed no abnormalities.

Electromyography gave a mildly diminished activity of the examined muscles; the palpebra sphincter (right and left), first inter-osseus dorsalis muscle (right) and anterior tibialis (right). After infra-orbital nerve stimulation, no R1 responses were received bilaterally. Retarded R2 responses were received, while the R2 response of the left side was significantly lower. Electroneurography indicated absence of an f wave when his right median, ulnar and peroneal nerves were stimulated. Absence of an f wave was also observed when his left tibial nerve was stimulated, indicating a mixed demyelinative polyneuropathy with a conduction blockade in multiple nerves and mild axial damage.

Our patient received 40 g intravenous Immunoglobulin for a duration of five days based on clinical suspicion. Notably, the CSF albumin became elevated (1 g/dL) only after the second lumbar puncture was performed two weeks after the onset of symptoms. Our patient received an additional 40 g of intravenous Immunoglobulin a few days later, for a duration of three days, in an attempt to ameliorate the final clinical outcome. Relapse can occur in patients who are treated early in the course of GBS and improve, and brief retreatment with the original therapy is usually effective in these cases [5,6]. On presentation with positive CMV IgM antibody test results, we applied an anti-CMV regime with ganciclovir intravenously 500 mg twice daily. The decision was taken to try to ameliorate our patient's neurological condition, which required him to remain resting in bed as any change in position caused inconvenience and a sense of falling. Intense physiotherapy was applied soon after diagnosis. Our patient markedly improved within two weeks. At that time, while still under anti-CMV treatment, he was able to sit up in bed and on a chair. Later on he was able to stand with support and also progressively take a few steps. After improvement of his clinical condition, our patient was discharged with appropriate follow-up.

## Discussion

Our patient presented to our emergency department using a wheelchair due to an inability to walk or stand up from a sitting position. The onset of symptoms was abrupt following an upper respiratory tract infection. Our differential diagnosis pointed to a type of GBS, which initially appeared with a sensory disturbance (hot and cold sensation) but was followed by a quite atypical descending progression, or other post-infectious neurologic diseases, including HIV, CMV, HSV, EBV, WNV, *C. jejuni*, *Haemophilus influenzae*, Lyme disease and TBC [7]. As suggested by Kaida *et al. *[8] in a recent review, the mechanism of action of infectious microorganisms that induce GBS may be attributed to the molecular mimicry of lipo-oligosaccharide genes that are responsible for the formation of human ganglioside-like lipo-oligosaccharide structures. Other causes, such as multiple sclerosis (MS) or other demyelinating syndromes, diabetes mellitus, B12 vitamin deficiency, drugs and chemical neuropathies were excluded following historical, clinical, laboratory and imaging tests. Examination for vasculitides, Sjögren's syndrome, sarcoidosis and paraneoplastic neuropathy proved negative.

Infection with CMV is the most common antecedent virus infection in GBS, as identified by the presence of IgM antibodies in 10% to 15% of patients at the onset of this disease. However, antiviral therapy is not classically recommended in GBS, as the disease is considered post-infectious. Recently, the presence of CMV DNA has been found in almost one-third of serum and CSF samples from patients with GBS who are positive for CMV-specific antibodies at the onset of the neurological disease. Furthermore, the time of lumbar puncture was critical for the detection of CMV DNA, as the probability of the presence of CMV DNA decreased significantly following an increase of the interval between onset of GBS and puncture. This association suggests that even more patients may carry CMV in CSF at a very early stage of GBS [9]. These findings indicate a link between neurological disease and CMV infection, but the clinical relevance remains to be elucidated.

The presence of herpes virus DNA in post-mortem tissue of patients suffering from epilepsy provides strong correlative evidence that this pathogen plays an important role in seizure circuits in the brain. In animal models, HSV-1 infection via the corneal route results in infection of the trigeminal ganglia and the transport of the virus into the central nervous system. At the neuronal level, HSV-1 infection causes a long-lasting reduction in the depolarizing membrane potential, thus resulting in a hyperexcitable neuronal state [10]. Like HSV-1, WNV has a significant propensity for infecting the limbic region, especially the hippocampus. Depending on the strain of WNV, infection in mice may or may not cause necrotic lesions with focal hemorrhages [11].

## Conclusions

In our patient, the clinical manifestation of GBS followed a combined herpes virus infection (CMV, EBV, HSV). Cross-reactivity between human herpes viruses has been suggested in a recent study in which the EBV virion glycoprotein gp85 was immunopreciptated by antisera to HSV-1, HSV-2 and CMV. Moreover, antisera to CMV and EBV neutralized the infectivity of both HSV-1 and HSV-2 at high concentrations, suggesting that cross-reactivity between these human herpes viruses may have pathogenic as well as evolutionary significance [12]. Performing an immunoprecipitation technique or immunoabsorption study using glycolipids or glycoproteins purified from EBV or CMV from patient serum samples would provide evidence for supporting the cross-reactivity between human herpes viruses in the case of our patient, but unfortunately these methods are not available in our hospital. Moreover, examination for anti-ganglioside antibodies was suggested to our patient in order to accommodate the diagnosis of GBS, but he did not give permission because the test was not available locally and consequently our patient would have had to pay for it privately.

This is an unusual clinical manifestation of GBS that initially appeared with a sensory disturbance (loss of cold and hot sensation) and followed a rather atypical descending clinical progress. As shown in Table 2, CMV IgM and IgG test results were positive on admittance and afterwards remained positive. However, EBV IgM test results were positive on admittance but became negative. The EBV IgG result was borderline on admittance and later remained positive. Our patient showed seroconversion at an early stage of EBV IgM to IgG antibodies, suggesting that EBV had been the cause of this syndrome. From our clinical and laboratory findings, we conclude that the method of seroconversion of EBV and CMV is very important. Even if this case is not the first report of this kind in the literature, we believe that it may contribute to the better understanding of the disease and the cross-reaction mechanisms of herpes virus infections.

## Consent

Written informed consent was obtained from the patient for publication of this case report and any accompanying images. A copy of the written consent is available for review by the Editor-in-Chief of this journal.

## Competing interests

The authors declare that they have no competing interests.

## Authors' contributions

FN, MT and KK analyzed and interpreted the data from our patient regarding the unusual clinical manifestation of the condition. AE and AA were consulted regarding the GBS and the viral contribution to the clinical manifestation, respectively. FN, AA and KK contributed to writing the manuscript. All authors read, edited and approved the final manuscript.
